# Development and Validation of the German Version of the Modified COVID-19 Yorkshire Rehabilitation Scale (C19-YRSm)

**DOI:** 10.3390/healthcare13151802

**Published:** 2025-07-24

**Authors:** Petra Lücker, Celine Bahr, Anika Kästner, Anke Steinmetz, Winfried Rief

**Affiliations:** 1 Physical and Rehabilitative Medicine, Clinic and Polyclinic for Orthopaedics, Trauma Surgery and Rehabilitative Medicine, University Medicine Greifswald, 17475 Greifswald, Germany; 2 Department of Psychology, Clinical Psychology and Psychotherapy, Philipps University Marburg, 35032 Marburg, Germany; 3 Section Epidemiology of Health Care and Community Health, Institute for Community Medicine, University Medicine Greifswald, 17487 Greifswald, Germany

**Keywords:** post-COVID condition, long COVID, C19-Yorkshire Rehabilitation Scale (modified), C19-YRS(m), translation, monitoring long COVID

## Abstract

**Background**: As a disease with a still largely unknown course, post-COVID requires a comprehensive multidimensional perspective and structured monitoring, as offered by the C19-YRSm. There has not yet been a German version of the scale. **Methods**: After the translation of the COVID-19 Yorkshire Rehabilitation Scale (modified version, C19-YRSm) into German, we conducted an online survey with it between 23 May 2023 and 10 May 2024 for patients with post-COVID condition. Participation took place twice; people received either only the German version or both the German and the original English versions of the scale at one-week intervals. Based on the results, reliability and validity of the German version of the C19-YRSm were extensively tested. **Results**: Data of 414 participants were analysed, 156 of whom took part twice at least seven days apart. Cronbach’s alpha coefficients of the subscales of the German version ranged from 0.75 to 0.93. In the English version, it ranged from 0.82 to 0.93. All the subscales correlate with each other with *p* < 0.001. For the overall scale and for three of the four subscales, the intraclass coefficients, as a measure of the agreement between measurement results at two points in time, showed good consistency. **Conclusions**: This study confirmed the reliability, applicability, and clinical utility of the C19-YRSm, aligning with previous studies. With its multidimensional structure and excellent quality criteria, the C19-YRS is a valuable asset for clinical practice and research. The validated German C19-YRSm holds significant potential to facilitate tailored interventions, destigmatise post-COVID conditions, and enhance patient care in medical contexts.

## 1. Introduction

After the SARS-CoV-2 pandemic, as of 13 May 2025, around 777.745 million people worldwide have been infected with the SARS-CoV-2 virus, and approximately 7.056 million people have died in association with the virus [1]. Some individuals experience persistent symptoms, defined as post-COVID-19 condition (PCC), which requires appropriate measuring instruments to assess the multidimensional nature of these heterogeneous conditions. The most commonly reported symptoms to date include fatigue, post-exertional malaise, cognitive impairments (such as brain fog, loss of concentration, and memory issues), sleep disorders, shortness of breath, anxiety and depression, chest pain, altered smell, persistent cough, and muscle pain [2,3,4,5]. Although some symptoms seem to decrease over time, most studies report that symptoms persist in the majority of patients for six months or longer [5,6,7,8].

Comprehensive and multidimensional questionnaires are important for diagnosing the post-COVID-19 condition. To date, existing questionnaires include the Post-COVID-19 Functional Status Scale (PCFS, [9]), the Symptom Burden Questionnaire™ for Long COVID (SBQ™-LC, [10]), the Post-COVID-Syndrome Score (PCS, [7]), and the COVID-19 Yorkshire Rehabilitation Scale (C19-YRS, [11]). These questionnaires assess symptoms of PCC, functional capability, and the impact of PCC on daily life.

The C19-YRS is one of the tools designed to capture a wide range of symptoms, including key symptoms, functional limitations, other symptoms, and overall health. It also assesses the impact of PCC on employment and incorporates the perspectives of close contacts, such as partners or caregivers. As it addresses all components of the ICF framework by the WHO, the C19-YRS is well-suited for assessing important aspects of the biopsychosocial status of an individual in medical contexts [12]. Furthermore, it has demonstrated good psychometric properties, including content and construct validity as well as good internal consistency [11,12].

Since 2022, a modified version of the C19-YRS (C19-YRSm) has been available, which has a smaller response scale than the C19-YRS, making it more intuitive for patients and enabling the effects of intervention to be recorded. Compared to the PCFS scale and the PCS score, the C19-YRSm covers a broader range of symptoms while being more concise than the SBQ™-LC [7,9,10]. This compactness is particularly suitable for patients with cognitive dysfunctions. Another key advantage of the C19-YRSm is its ability to capture both current symptoms and those present prior to SARS-CoV-2 infection. This allows us assess the condition according to the WHO and National Academies of Sciences, Engineering, and Medicine (NASEM) definition, identifying new or worsening symptoms [13,14].

There are different translations of the C19-YRSm, e.g., English, Spanish, and Turkish [12,15,16]. Given the advantages of the C19-YRSm and the need for a multidimensional tool for assessing the broad range of post-COVID-19 symptoms, it is highly desirable to make the C19-YRSm accessible in German-speaking countries. In particular, we expect significant benefits from using the C19-YRSm in medical contexts, both for enabling suitable interventions for individual patients and for reducing stigma for those affected. We hypothesise that the German translation will assess the symptoms with comparable validity and exhibit similar psychometric properties as the original instrument. Here, we present results of a translated and validated German version of the C19-YRSm.

## 2. Materials and Methods

The validation process was carried out in two steps. In the first step, the scale was translated into German according to Beaton et al. [17], and in the second step, we conducted an online survey for validation. Based on the COSMIN Study Design Checklist for Patient-Reported Outcome Measurement Instruments [18], we reported on the translation and validation of the German version of the C19-YRS.

### 2.1. Instrument

The modified COVID-19 Yorkshire Rehabilitation Scale (C19-YRSm) [12] is a 17-item patient-reported outcome measure (PROM) for assessing and monitoring PCC, which has been widely used in the United Kingdom [19].

The instrument is divided into four subscales:

(1) Symptom Severity (10 items, 26 variables, score range of 0–30, with 30 representing the most severe manifestation of symptoms).
(2) Functional Ability (5 items, 5 variables, score range of 0–15, with 15 representing the most severe limitations of abilities).
(3) Other Symptoms (1 item, 25 variables + 1 open text, score range of 0–25, with 25 representing the highest number of symptoms reported).
(4) Overall Health (1 item, 1 variable, score range of 0–10, with 10 representing the best health).

The differences between items and variables mentioned in the first two subscales result from the fact that, for example, the item ‘breathlessness’ asks specifically for (a) at rest; (b) changing position, e.g., from lying to sitting or sitting to lying; (c) on dressing yourself; and (d) on walking up a flight of stairs. The worst value out of these four variables is the score for this item.

The subscales Symptom Severity and Functional Ability are assessed using a 4-point Likert scale from 0 to 3. Zero indicates no symptoms, 1 indicates a mild problem (that does not interfere with daily life), 2 indicates a moderate problem (that interferes with daily life to some degree), and 3 indicates a severe problem (that interferes with life or all aspects of daily life). The items’ scores are summed up within the scales. For the scores of the subscales Symptom Severity and Functional Ability, all items must be answered. As a result, the total score of the C19-YRSm can range from 0 to 80, with higher values indicating a stronger manifestation of PCC. For scales 1, 2, and 4, there are corresponding scales for the time before COVID-19 to assess pre-illness health. The entire scale is used as a self-report instrument.

### 2.2. Translation Process and Resulting Questionnaire

After obtaining permission to use and translate the scale from the University of Leeds, the translation and transcultural adaptation of the C19-YRSm was performed in five steps [17]:

1. Translation—2. Synthesis—3. Back translation—4. Expert Committee review—5. Pre-testing.

In the first step, two bilingual persons translated the C19-YRSm independently from each other from English into German (forward translation). One of them had a nursing and health research background (P1), and the other person was an English language student (P2).

The second step involved the two translators (P1 and P2) discussing the two German versions and agreeing on a common version with another researcher, who acted as a recorder. In the next step, two other persons individually translated the German version back into English (back translation) for validity checking. This procedure is intended to ensure that the translated version has the same content as the original version.

One of the back translators was a native English speaker living in Germany (B1), and the other was a German native speaker who has lived in the United Kingdom for several years (B2). In a fourth step, an expert committee, which consisted of the two English–German translators (P1 and P2), the two German–English translators (B1 and B2), a methodologist, and an experienced medical doctor, discussed the translated versions. A provisional German version was agreed on the basis of the versions of the instrument produced up to that point.

To carry out the pre-test, the survey was implemented in the web-based tool SoSci Survey [20]. The German version of the questionnaire was completed online by 22 participants with the option to comment on every single item as well as on the entire questionnaire. This sample was recruited from personal or professional contacts of the researchers and consisted of four men and 18 women with a mean age of 43 and 45 years, respectively (SD = 16, 17). The aim of the pre-test was to check the questionnaire for general comprehensibility and to make suggestions for improvement. We decided to include people with and without ongoing symptoms after a SARS-CoV-2 infection. This was mainly because anyone can be affected by the disease, regardless of, e.g., age or gender, and the pre-test primarily focused on the comprehensibility of terms and formulations.

### 2.3. Modifications in the German Version

The comments from the pre-test were discussed in the research team, and changes were implemented accordingly. In addition to confirming good comprehensibility and pointing out some spelling mistakes, the comments also included suggestions for improvement. Some were suggestions for minor changes to the wording but also for fundamental changes to the scale itself, which could not be considered. The comments we were able to consider were, for example, the possible interpretability of the meaning of the term ‘now’ in the table heading. For clarification, we added ‘in the last 7 days’ in brackets.

In the symptom severity and functioning subscales, the response option ‘don’t know’ was added for the information on symptoms that already existed before infection with SARS-CoV-2. In the German version, the instructions for completing the subscale are repeated above the second subscale.

In the original version of the C19-YRSm, in the subscale Other Symptoms, the presence of various current and pre-existing symptoms that worsened after the infection is asked for in one item. It is only to be ticked if the respective symptom is present. The suggestion of one of the pre-testers to ask for new and worsened symptoms in two items was implemented. Thereby, in the German version, a change in symptoms before and after the infection can be assessed additionally.

In addition, we added the item ‘Already retired before the illness to the German version in order to obtain a complete picture of the effects of PCC on the employment status.

### 2.4. Study Design

This study design was a prospective longitudinal study.

For the validation of the adapted German version of the C19-YRSm, the survey was again implemented in the web-based tool SoSci Survey [20]. The sample size calculation was based on the study of Gorsuch et al. [21], who recommended including at least ten participants per item of the C19-YRSm. Therefore, the aim was to recruit a total of 170 participants.

Participants were asked to complete the survey twice (T1; T2) within approximately one week. They were randomly assigned to one of three groups: (a) German–German (50 %), (b) English–German (25%), and (c) German–English (25%). The assignment was based on the time of receipt of the e-mails. The e-mail addresses were copied into four different lists, which stood for the different combinations of questionnaires (T1_German-T2_German, T1_German-T2_English, T1_German-T2_German, T1_English-T2_German). The German–German combination was created twice, first in order to have groups of equal size and second in order to achieve 50% monolingual and 50% bilingual completion of the questionnaire. In this way, a structured but nonetheless randomised assignment was made. Participants each received two different links to the four surveys (T1_German, T1_English and T2_German, T2_English) at one-week intervals.

The participants themselves generated a pseudonym for participation, which consisted of the first two letters of their first name, the first two numbers of their date of birth, and the first two letters of their mother’s name. This pseudonym made it possible to match the questionnaires of the two survey time points, T1 and T2.

In the first survey (T1), socio-demographic information, such as age, sex, or school education, was obtained, as well as data on infections with SARS-CoV-2 and vaccinations against the disease. In the second survey (T2), only the C19-YRSm was provided. At the end of both questionnaires, participants were also offered the opportunity to comment on the survey.

### 2.5. Ethics

This study protocol was reviewed and approved by the Ethics Committee of the University Medicine Greifswald (No. BB 015-23) in accordance with the applicable legal regulations and the Declaration of Helsinki in its current version. This study was prospectively registered in the German Clinical Trials Registry (DRKS00031587, 4 May 2023). Participants were informed about the purpose of this study on an information page. By requesting to receive questionnaire links, they declared their participation in this study.

### 2.6. Recruitment and Inclusion Criteria

Participants were recruited from all over Germany, e.g., via social media (such as Facebook, Twitter/X, and Instagram) and via patient initiatives such as Long COVID Deutschland (Long COVID Germany) and NichtGenesen (“NotRecovered”), as well as via hospitals and rehabilitation clinics.

First, those interested in this study were directed to an information page explaining its purpose and providing the research team’s email address, which could be used to request participation in this study.

Inclusion criteria were (a) ≥16 years of age, (b) the declaration of at least one infection with SARS-CoV-2, (c) reporting persistent new or increased symptoms after the infection, and (d) the specification of a pseudonym in order to be able to exclude multiple participations at the same time. A positive antigen or antibody test was not required, as routine tests were no longer carried out at the time of the survey. We requested participants to have a basic knowledge of English to ensure they could complete the English questionnaire if assigned to it. If participants stated, after receiving the links, that they were unable to complete the English questionnaire, they did not receive an additional link to the German version. We did not ask for the pseudonym given but assumed that the questionnaire was incomplete with regard to the C19-YRSm scales and was therefore automatically excluded.

### 2.7. Data Preparation and Statistical Analysis

First, the pseudonym was used to check for repeated participation, i.e., participants opened the link and answered the questionnaire more than once. For these cases, pseudonyms and other information were compared manually, and cases with less information were excluded.

The scores of the scales and subscales of the C19-YRSm were each calculated as specified by the developer (see Instrument). The one-item scale ‘Overall health’ was inverted to align with the direction of other scales (i.e., higher values indicate worse overall health).

The interval between the first and second participation was set to at least seven days, resulting in distinct data sets for participation at time 1 (T1) and participation at time 2 (T2) (distinguishable by the different links that were used) and a data set merged from both, comprising participants who completed both assessments.

For cross-sectional analysis, sociodemographic data and the psychometric properties of the C19-YRSm scale and its subscales were compared between participants who completed the German version and those who completed the English version of the C19-YRSm. Metrics such as the mean with the standard deviation (SD), the median with the interquartile range (IQR), and the minimum and maximum inter-item correlations were calculated for both the overall scale and the subscales. For descriptive analysis of metric variables, the *t*-test was applied, which is robust even when the normality assumption is violated [22], and for categorical variables, the Chi-square test was used. Internal consistency was assessed using Cronbach’s alpha for both the overall scale and the subscales. Moreover, Cronbach’s alpha was calculated for the deletion of single items within scales. However, Cronbach’s alpha was not calculated for the Overall Health scale, as it consists of only a single item.

Longitudinal analysis included only participants who completed the questionnaire twice. Psychometric properties of the C19-YRSm scale and subscales were compared between T1 and T2, irrespective of the language version used. For both the overall scale and subscales, metrics such as the mean with SD, median with IQR, range of item means, range of item SDs, minimum and maximum inter-item correlations, and Cronbach’s alpha were reported. Additionally, Spearman’s rank correlation coefficients with 95% confidence intervals were computed for each subscale.

Thereafter, participants who completed the questionnaire twice were further divided into two subgroups:

- The monolingual group, comprising participants who completed the C19-YRSm twice in German (German–German).
- The bilingual group, comprising participants who completed the C19-YRSm in both English and German (either English–German or German–English).

The participants in the bilingual group were not bilingual in the strict sense. They were only able to complete the questionnaire in both languages due to their English language skills, e.g., English learnt at school.

For the overall scale and each of the subscales, the mean and SD were calculated separately for T1 and T2. Differences between T2 and T1 were computed for each scale and subgroup, and the mean T2-T1 differences were compared between the monolingual and bilingual groups using an independent *t*-test.

Test–retest reliability was evaluated using the intraclass correlation coefficient (ICC) with its 95% confidence intervals. Reliability was classified as moderate (ICC: 0.5–0.75), good (ICC: 0.75–0.9), or excellent (ICC > 0.9) based on the criteria established by Koo et al. [23]. Comparisons of ICCs between the monolingual and bilingual subgroups were conducted using Fisher’s z-transformation [24].

When data in the subscales Symptom Severity or Functional Ability were missing, the subscales were excluded from analyses. This was the case in less than 2% of cases within the subscales (0.4–1.9%).

The data were analysed using the Statistical Package for Social Sciences (SPSS, Version 29), and statistical significance was set at a two-sided *p*-value of <0.05.

## 3. Results

The survey was conducted from 23 May 2023 to 31 December 2023. As a first analysis showed that the sample size of participants who had completed the questionnaire twice, as planned, was not sufficient for analysing test–retest reliability, the survey was reopened from 1 February 2024 to 10 May 2024. Recruitment was again carried out via social media and interest groups.

The two links were sent to 578 people, of whom 505 provided at least some information. We included the data of 414 respondents (T1) in the analyses. A total of 91 participants had to be excluded because they did not provide a pseudonym (*n* = 31), and therefore, multiple participation could not be ruled out; they did not answer any of the items of the C19-YRSm (*n* = 30); they indicated they were affected by post-vac (*n* = 25); they were younger than 16 years (*n* = 4); or they completed the questionnaire twice (*n* = 1).

### 3.1. Patient Characteristics

Of the 414 participants of T1, *n* = 323 completed the questionnaire in German and *n* = 91 in English. Additionally, *n* = 156 (37.7%) of the 414 participants in T1 completed the questionnaire a second time (T2) with an interval of at least seven days. Of those who took part twice, 125 completed the questionnaire twice in German (monolingual group), and 31 completed it once in German and once in English (bilingual group).

The characteristics of the participants are shown in Table 1.

No significant differences were found between the group who completed the questionnaire in German and that who completed it in English in terms of socio-demographic information like gender or age. The participants were on average 44.8 years old, and 84% of them were female. The time between the first infection with SARS-CoV-2 and participation in the survey ranged between 51 and 1400 days and was, on average, 574.56 days (approx. 1.5 years).

For the overall scale as well as for the subscales Symptom Severity, Functional Ability, Other Symptoms, and Overall Health, the mean values and the standard deviation and other values are shown in Table 2. The mean values were consistently lower in the group who completed the questionnaire in English, indicating that participants generally reported fewer and less severe symptoms.

The scales, apart from Overall Health, were also tested for internal consistency. For the group that completed the German questionnaire, Cronbach’s alpha coefficients ranged from 0.75 to 0.93, and for the group that completed the English version, it ranged from 0.82 to 0.93. In both groups, Functional Ability had the lowest Cronbach’s alpha (Table 2) but still an acceptable level of internal consistency.

No significant differences between the two groups were detected regarding the overall C19-YRSm score, the symptom severity, the functional ability, and other symptoms. However, participants in the group who completed the questionnaire in English reported slightly better overall health (German: 2.96; English: 3.58; *p*-value = 0.028). However, this group’s being healthier can only be understood as a trend.

Cronbach’s alpha would not increase considerably in any of the subscales by deleting items. The difference was not greater than 0.078.

When using histograms, no floor or ceiling effects were found in either the German or the English group.

### 3.2. Longitudinal Analysis

Overall, N = 156 participants took part in the survey twice within at least 7 days, either by completing only the German questionnaire (monolingual group, *n* = 125) or by completing both the German and the English questionnaires (bilingual group, *n* = 31).

Analyses of the data, separated according to the two points in time of participation, are listed in Table 3.

The subscales Symptom Severity and Functional Ability have a moderate positive correlation (r ≥ 0.4) with all the other subscales; only the correlation between Other Symptoms and Overall Health is weaker (r < 4). However, all the subscales correlate with each other with *p* < 0.001.

For reliability analysis, Cronbach’s alpha was calculated to assess the internal consistency of the main subscales Symptom Severity (10 items and 26 variables) and Functional Ability (5 items and 5 variables). The internal consistency of the subscales was satisfying, with Cronbach’s alpha for Symptom Severity = 0.90 and 0.92 and for Functional Ability = 0.77 and 0.82.

The Other Symptoms subscale only indicates the presence of symptoms. As these are independent of each other, Cronbach’s alpha was not calculated. For the total C19-YRSm, Cronbach’s alpha is 0.92 (T1) and 0.93 (T2).

### 3.3. Test–Retest Reliability

For those who participated twice (*N* = 156), the test–retest reliability between T1 and T2 was evaluated by intraclass correlation coefficient (ICC) and its 95% confidence interval (Table 4).

For the overall sample, the mean values of the overall scale and the subscales were lower at T2 than at T1. The smallest difference was found in the subscale Overall Health.

Separated by mono- and bilingual participation, the mean values of the scales were lower at T2. The only exception was the general state of health, which was contradictory in the bilingual group: At T2, the scale showed higher values on average, and thus indicated a poorer state of health.

For the C19-YRSm overall scale and three of the four subscales, the intraclass coefficients as a measure of the agreement between measurement results at two points in time showed good consistency [25]. They were generally higher in the monolingual group than in the bilingual group.

However, for the one-item subscale Overall Health, the ICC in the overall sample is lower but acceptable. Separated by mono- and bilingual participation, the ICC in the bilingual group is very low.

Five people rated their overall health within a week with a difference of five or more points on an 11-point scale.

The overall differences between T1 and T2 were not clinically significant or did not show significant improvements or worsening of the disease within seven days, although this may apply for individual participants, as they reported considerable differences in their overall health.

### 3.4. Further Details

Participants noted repeatedly that the option ‘no’ or ‘not existing’ should be offered for the subscale Other Symptoms.

## 4. Discussion

With this study, we were the first to translate the English version of the C19-YRSm into German and to validate it successfully. We were able to confirm its good reliability and validity criteria. The internal consistency of the subscales was satisfying, with Cronbach’s alpha above 0.77 for the subscales and Cronbach’s alpha 0.92 (T1) and 0.93 (T2) for the total C19-YRSm. Modifications were made to the German version to enhance comprehensibility. In summary, the German version was generally rated as easy to understand in the pre-test, and it was considered highly comparable to the English version.

Adapting the questionnaire to the German cultural context was relevant to support its validity and improve comprehensibility and comparability while minimising misunderstandings and distortions. Additionally, it aligns with the German healthcare system and relevant research fields, and there are no patient-reported instruments available in the German-speaking context to measure such a wide range of different symptoms connected with post-COVID-19. It also aligns with the results of former translations, which also show good internal consistency and reliability [15,16]. The German version performs at least as well as the other versions.

The use of the C19-YRS in German health care contexts could facilitate tailored interventions for individual patients and contribute to the destigmatisation of those affected by PCC [26]. While the C19-YRSm is comprehensive and captures the broad and heterogeneous spectrum of symptoms [2], it is also applicable across various medical settings, where standardised instruments are essential for diagnostics and treatment planning [27].

Additionally, the scale addresses all components of the WHO’s International Classification of Functioning, Disability, and Health (ICF) framework [12]. The C19-YRSm also outshines other existing tools like the PCFS-Scale, PCS, and SBQ™-LC, especially with its ability to capture both current symptoms and those existing prior to SARS-CoV-2 infection. This functionality supports adherence to the WHO and NASEM definition of PCC by distinguishing between new symptoms and those that have worsened [13,14].

In addition to the important ability to capture both current and pre-SARS-CoV-2 symptoms, the C19-YRSm has further advantages, as it can be used in different settings. It is a multidimensional tool that measures both physical changes caused by the COVID-19 disease and resulting psychological changes. Therefore, the scale can be used for diagnosis in both medical and psychological settings. Patients with long COVID often need multidisciplinary treatment [27,28] and can therefore be found in multiple departments in order to receive support in dealing with the condition and its consequences. The scale can also be used in research contexts.

### 4.1. Interpretation of the Results

As previously mentioned, the internal consistency of the subscales was satisfying, suggesting that our translation is highly comparable to the original English version and aligns with data from previous translations, such as those by Acosta-Mari et al. [15] and Doğan et al. [16].

Ten participants in the bilingual group showed considerable differences in the sum between the two measurement points (T1 and T2, >3 points), with five of them showing differences of five or more points. This could also have contributed to the notably low ICC in the bilingual group. However, the corresponding correlation only showed a trend in this direction. Therefore, this does not appear to be a translation problem but may be due to considerable changes in the one-item scale, Overall Health. It might be that even small changes in severity or symptoms have a large impact on the subjective perception of overall health, which then influences the score of the C19-YRS(m) overall scale.

The comparison of results of the mono- and bilingual groups shows that the comparability of the versions is very good. No significant differences were identified between the German and English groups in socio-demographic characteristics such as gender and age. Similarly, the two groups did not differ significantly in overall C19-YRSm scores, symptom severity, functional ability, or other symptom measures. This confirms the comparability of the English and German versions. The calculated intraclass correlation coefficients (ICCs) also indicate good consistency [25].

In comparison to the translation studies conducted by Doğan et al. [16] and Acosta-Mari et al. [15], our study differs in its methodology. Our study involved a comparative analysis between the German version and the original English version based on two consecutive measurements, thereby allowing for the evaluation of test–retest reliability.

Regarding demographic variables, our participants appear to be comparable to those of Acosta-Mari et al. [15] in terms of gender, with a higher proportion of female participants. Since no specific information is provided about the average age of the participants in their study, our participants seem only comparable to those in this study by Doğan et al. [16], whose participants, however, were generally older than ours. As in the studies mentioned above, the majority of participants in our study also have a higher level of education.

With regard to the burden experienced by study participants, our findings suggest that, in contrast to Acosta-Mari et al.’s cohort [15], our participants exhibit a greater degree of burden on the subscales of Symptom Severity and Functional Ability. While the levels of both populations are comparable with respect to the subscale of Other Symptoms, the subscale of Overall Health indicates a higher perceived burden in our sample compared to that of Acosta-Mari et al. [15].

Due to the multiple comments that the answer option “no” or “not existing” should be offered in the Other Symptoms subscale, the following sentence was added to the instructions of the final questionnaire: ‘If you do not have a symptom, simply do not tick anything.’

### 4.2. Limitations of This Study

Recruitment turned out to be more difficult than expected, and the number of participants completing both questionnaires fell slightly below the originally planned sample size. The ratio of mono- and bilingual participation is not balanced, which resulted in the bilingual subgroup being underpowered for the calculation of the ICC. This may explain the lower ICC observed in the bilingual group for the ‘Overall Health’ subscale; however, it may also be attributable to the subscale’s single-item structure, which is more susceptible to measurement error and is often limited in its reliability [29]. It is possible that the English version of the questionnaire was more difficult to complete for some participants or that they were already involved in other studies due to the large number of long COVID studies available. Nevertheless, the analysis of test–retest reliability remained feasible. In future studies, the validity of the instrument should be further evaluated, and the results of studies using the German version of the questionnaire should be compared with studies using the English version.

A potential limitation of our study is that we did not verify an infection with SARS-CoV-2 or the diagnosis of PCC and relied on participants self-reports of symptoms. Therefore, it is possible that we included participants who did not meet official diagnosis criteria. However, at the outset of our study, obtaining such a diagnosis was challenging due to limited diagnostic capabilities at this time. Therefore, we opted not to require an official diagnosis to maximise the inclusion of patients who met the official WHO definition [13]. To ensure that included participants match the target population, future studies should incorporate standardised screening instruments or symptom-based criteria. This approach also reduces the risk of including individuals who merely self-report a diagnosis without formal clinical verification.

The majority of participants in this study were female and had higher educational levels. Nevertheless, it is known that the disease predominantly affects or is diagnosed in middle-aged women. The data in our study align well with existing profiles of PCC patients, as shown in other studies [30,31]. As higher educational levels could not be proven as a risk factor for PCC, further studies should aim to recruit a more heterogeneous sample.

At least, although adding complementary scales to measure key symptoms, functional limitations, other symptoms, and overall health would have been the gold standard, no sufficiently comparable validated scales were available for these constructs. This limits the assessment of convergent validity and should be addressed in future research to confirm the instrument’s capacity for multidimensional assessment. At the same time, it highlights the relevance of the C19-YRSm in this context.

## 5. Conclusions

In conclusion, as in former translations (e.g., [15,16]), the German translation of the C19-YRSm demonstrates strong reliability and usability, making it a valuable asset for clinical practice, rehabilitation, and research. It is highly comparable to the English original version. Its comprehensive assessment of symptoms, functional limitations, and biopsychosocial status underscores the importance of its accessibility in German-speaking countries. The validated German C19-YRSm holds significant potential to facilitate tailored interventions, destigmatise PCC, and improve patient care. In addition, the scale enables the severity of symptoms and impairments to be monitored and assessed over the course of the disease. This can be used to draw conclusions about successful therapies.

## Figures and Tables

**Table 1:** Characteristics of all participants (*N* = 414), stratified by language version of the C19-YRSm.

	German C19-YRSm Version (n = 323)	English C19-YRSm Version (n = 91)	*p*-Value
Sex, n (%)			0.154
- Male	42 (13.0)	19 (21.1)	
- Female	277 (85.8)	70 (77.8)	
- Diverse	4 (1.2)	1 (1.1)	
Age (*M (SD*))	45.33 (11.92)	42.97 (13.10)	0.102
Education, n (%)			
- Still at school	5 (1.5)	1 (1.1)	
- 8–9 years of school education	8 (2.5)	5 (5.5)	
- 10 years of school education	99 (30.7)	25 (27.5)	
- >10 years of school education	206 (63.7)	60 (66.0)	
- Without a school-leaving certificate	3 (0.9)	-	
- Other	2 (0.6)	-	
Time (in days) between first infection and (first) participation in the survey (*M (SD*))	573.25 (281.44)	579.31 (259.14)	0.877
Missing, n (%)	92 (28.5)	27 (29.7)	
Presence of persistent symptoms after first infection (*N* = 401) (yes/no, n (%))	289 (92.3)	80 (90.9)	0.824
Missing incl. ‘cannot remember’, n (%)	10 (3.1)	3 (3.3)	
Vaccination against COVID-19, n (%)			
- Not vaccinated	20 (6.2)	4 (4.4)	
- 1× vaccinated	15 (4.6)	5 (5.5)	
- 2× vaccinated	67 (20.7)	21 (23.1)	
- 3× vaccinated	175 (54.2)	49 (53.8)	
- 4× vaccinated	42 (13.0)	11 (12.1)	
- >4× vaccinated	4 (1.2)	1 (1.1)	

*M* = mean; *SD* = standard deviation.

**Table 2:** Psychometric properties of the overall C19-YRSm scale and subscales (N = 414, one-time participation (T1)).

	German (n = 323)	English (n = 91)	*p*-Value
**C19-YRSm (0–80)**			0.121
Mean (SD)	43.7 (12.32)	41.3 (12.67)	
Median (IQR)	44 (17)	41 (16)	
Inter-item correlations (minimum-maximum)	0.087–0.710	−0.060–1.000	
Cronbach’s alpha	0.93	0.93	
Missing N (%)	6 (1.9)	7 (7.7)	
**Symptom Severity (0–30)**			0.205
Mean (SD)	19.68 (5.13)	18.78 (6.18)	
Median (IQR)	20 (7)	20 (9)	
Inter-item correlations (minimum-maximum)	0.158–0.711	0.154–0.731	
Cronbach’s alpha	0.91	0.93	
Missing N (%)	1 (1.1)	1 (1.1)	
**Functional Ability (0–15)**			0.212
Mean (SD)	8.99 (3.20)	8.43 (3.87)	
Median (IQR)	9 (4)	9 (5)	
Inter-item correlations (minimum-maximum)	0.065–0.317	0.301–0.436	
Cronbach’s alpha	0.75	0.82	
Missing N (%)	2 (0.6)	1 (1.1)	
**Other Symptoms (0–25)**			0.103
Mean (SD)	7.75 (5.26)	6.76 (4.66)	
Median (IQR)	7 (7)	6 (7)	
Inter-item correlations (minimum-maximum)	−0.042–0.316	−0.026–0.389	
Cronbach’s alpha	0.86	0.82	
Missing N (%)	2 (0.6)	0 (0.0)	
**Overall Health (0–10)**			0.028
Mean (SD)	2.96 (1.84)	3.58 (2.43)	
Median (IQR)	3 (2)	3 (3)	
Missing N (%)	4 (1.2)	3 (3.3)	

**Table 3:** Psychometric properties of the C19-YRSm subscales (*N* = 156, two-time participation).

	Symptom Severity (0–30 Points)		Functional Ability (0–15 Points)		Other Symptoms (0–25 Points)		Overall Health (0–10 Points)	
	T1	T2	T1	T2	T1	T2	T1	T2
Mean (SD) *p*-value	19.32 (4.99)	17.97 (5.55)	9.06 (3.33)	8.38 (3.44)	7.41 (4.73)	7.01 (4.59)	6.86 (2.04)	6.90 (1.95)
	<0.001		0.001		0.105		0.907	
Median (IQR)	20 (7)	18 (8)	9 (4)	8 (5)	7 (8)	6 (7)	3 (2)	3 (2)
Item mean range	0.43–2.70	0.39–2.49	1.01–2.51	0.86–2.36				
Item SD-range (minimum-maximum)	0.63–1.09	0.69–1.05	0.74–1.00	0.78–1.00				
Inter-item correlations (minimum-maximum)	0.124–0.643	0.151–0.718	0.220–0.290	0.267–0.397				
Cronbach’s alpha	0.90	0.92	0.77	0.82				
**Subscale correlations (Spearman-Rho)**								
**Symptom Severity** r (*p*-value) CI	-	-	0.667 (<0.001) 0.566–0.748	0.675 (<0.001) 0.575–0.755	0.594 (<0.001) 0.479–0.690	0.548 (<0.001) 0.423–0.652	0.466 (<0.001) 0.329–0.584	0.435 (<0.001) 0.293–0.558
**Functional Ability** r (*p*-value) CI			-	-	0.518 (<0.001) 0.389–0.628	0.419 (<0.001) 0.275–0.545	0.545 (<0.001) 0.421–0.650	0.631 (<0.001) 0.522–0.720
**Other Symptoms** r (*p*-value) CI					-	-	0.307 (<0.001) 0.153–0.446	0.265 (<0.001) 0.107–0.410

**Table 4:** Scores on the C19-YRSm scale and subscales for the overall sample and monolingual and bilingual subgroups with a comparison of T1 versus T2.

Overall Sample N = 156				Monolingual N = 124				Bilingual N = 31				Monolingual vs. Bilingual	
T1 *m (SD)*	T2 *m (SD)*	T2-T1 *m (SD)*	ICC (95% CI) *p*-Value	T1 *m (SD)*	T2 *m (SD)*	T2-T1 *m (SD)*	ICC (95% CI) *p*-Value	T1 *m (SD)*	T2 *m (SD)*	T2-T1 *m (SD)*	ICC (95% CI) *p*-Value	*p*-Value of T2-T1	*p*-Value of ICC
**C19-YRS(m) overall scale**													
43.37 (11.17)	41.39 (11.24)	−1.82 (6.02)	0.86 (0.81, 0.90) <0.001	43.76 (11.30)	41.53 (11.30)	−2.15 (5.38)	0.89 (0.84, 0.92) <0.001	41.73 (10.67)	40.87 (11.19)	−0.50 (8.03)	0.73 (0.51, 0.86) <0.001	0.571	0.024
**Symptom Severity**													
19.32 (4.99)	17.97 (5.55)	−1.11 (3.31)	0.71 (0.63, 0.78) <0.001	19.47 (4.49)	17.82 (5.71)	−1.35 (2.98)	0.73 (0.64, 0.80) <0.001	18.71 (5.41)	18.55 (4.92)	−0.16 (4.28)	0.66 (0.40, 0.82) <0.001	0.331	0.441
**Functional Ability**													
9.06 (3.33)	8.38 (3.44)	−0.54 (2.00)	0.77 (0.81, 0.90) <0.001	9.17 (3.27)	8.34 (3.58)	−0.62 (1.84)	0.80 (0.72, 0.85) <0.001	8.61 (3.60)	8.55 (2.87)	−0.23 (2.53)	0.67 (0.42, 0.83) <0.001	0.401	0.242
**Other Symptoms**													
7.41 (4.73)	7.01 (4.59)	−0.33 (2.77)	0.83 (0.77, 0.87) <0.001	7.50 (4.73)	7.06 (4.51)	−0.41 (2.71)	0.84 (0.77, 0.88) <0.001	7.06 (4.80)	6.81 (4.98)	−0.03 (3.03)	0.79 (0.61, 0.89) <0.001	0.984	0.497
**Overall Health**													
6.86 (2.04)	6.90 (1.95)	−0.03 (1.67)	0.47 (0.33, 0.58) <0.001	6.98 (1.88)	6.88 (2.07)	−0.13 (1.47)	0.53 (0.39, 0.65) <0.001	6.39 (2.58)	6.97 (1.38)	0.37 (2.28)	0.25 (−0.11, 0.55) 0.088	0.447	0.162

*m*—Mean; *SD*—Standard Deviation; CI—Confidence Interval; ICC—Intraclass Correlation Coefficient.

## Data Availability

Data available on reasonable request due to restrictions (legal reasons).
